# Exacerbation of Hyperparathyroidism, Secondary to a Reduction in Kidney Function, in Individuals With Vitamin D Deficiency

**DOI:** 10.3389/fmed.2020.00221

**Published:** 2020-06-05

**Authors:** Makoto Daimon, Tomoyuki Fujita, Masaya Murabayashi, Satoru Mizushiri, Hiroshi Murakami, Yuki Nishiya, Jutaro Tanabe, Yuki Matsuhashi, Miyuki Yanagimachi, Itoyo Tokuda, Kaori Sawada, Kazushige Ihara

**Affiliations:** ^1^Department of Endocrinology and Metabolism, Hirosaki University Graduate School of Medicine, Hirosaki, Japan; ^2^Department of Oral Healthcare Science, Hirosaki University Graduate School of Medicine, Hirosaki, Japan; ^3^Department of Social Medicine, Hirosaki University Graduate School of Medicine, Hirosaki, Japan

**Keywords:** CKD, PTH, vitamin D, population based study, environmental interaction

## Abstract

**Aims/Introduction:** Chronic kidney disease (CKD)-mineral and bone disorders (CKD-MBD) are an adverse outcome derived from decreases in kidney function, where abnormality of serum concentrations of calcium (Ca), phosphorus, parathyroid hormone (PTH), and vitamin D can be seen simultaneously. To identify individuals at risk for CKD-MBD or secondary hyperparathyroidism, the relationships between estimated glomerular filtration rate (eGFR) and serum PTH concentration were evaluated, allowing for confounding factors, in particular vitamin D status, in a general Japanese population.

**Materials and Methods:** Nine-hundred-and-thirty participants in the population-based Iwaki study conducted in 2016 who were not on drugs affecting mineral metabolism nor hemodialysis, were included in the study (326 men and 604 women; age: 55.4 ± 15.9 years).

**Results:** Regression analysis showed a significant correlation between eGFR and serum intact PTH concentration, after adjustment for possible confounding factors (β = −0.122, *p* < 0.001). The smoothed spline curve applied for the correlation analysis revealed a biphasic correlation, with a division at an eGFR of ~60 mL/min/1.73 m^2^, below which the correlation coefficient was higher (β = −0.405, *p* < 0.001). Stratification on the basis of vitamin D status showed that the correlation was present only in participants with vitamin D deficiency (25-dihydroxyvitamin D3: <15 pg/mL) (β = −0.154, *p* < 0.001).

**Conclusions:** These results indicate that a reduction in eGFR is a significant risk factor for an increase in serum PTH concentration when it is <60 mL/min/1.73 m^2^ and vitamin D is deficient, in the general Japanese population.

## Introduction

Chronic kidney disease (CKD) is a worldwide public health issue, because CKD is a risk not only for end-stage kidney disease (ESKD), but also for CKD-related mineral and bone disorders (CKD-MBD), as well as cardiovascular events and ultimately mortality (1–4). CKD-MBD is a syndrome characterized by dysregulation of minerals and bone metabolism, bone fragility, and vascular calcification, each of which has been shown to be associated with greater morbidity and mortality in studies of CKD (5–7). Therefore, the prevention of CKD-BMD potentially is an effective means of preventing CKD-related increases in morbidity and mortality.

Abnormal serum concentrations of calcium (Ca), inorganic phosphorus (InP), parathyroid hormone (PTH), and vitamin D are features of the dysregulation of mineral and bone metabolism, because the kidney precisely regulates serum Ca and InP concentrations by regulating the activation of vitamin D and the serum concentration of PTH. Specifically, as CKD progresses, the activation of vitamin D decreases, resulting in hypocalcaemia and consequently secondary hyperparathyroidism, which in turn stimulates bone osteoclast activity and generates bone abnormalities.

Therefore, the prevention of secondary hyperparathyroidism or an increase in serum PTH concentration is recommended to ameliorate the bone abnormalities present in patients with ESKD (7, 8). Furthermore, subclinical changes in bone metabolism have been reported as early as stage 2 of CKD (9–12). For that reason, therapy aimed at lowering the serum concentration of PTH may be useful, even in patients in the earlier stages of CKD, although the effectiveness of such therapy has not been thoroughly assessed. In addition, serum PTH concentration is also known to increase when vitamin D status is suboptimal (13–15). Consequently, the increase in serum PTH concentration induced by renal dysfunction may depend at least partially on vitamin D status.

In the present study, we evaluated the relationships between estimated glomerular filtration rate (eGFR) and serum PTH concentration, allowing for confounding factors, in particular vitamin D status, to identify individuals at risk for CKD-MBD or secondary hyperparathyroidism in a general Japanese population.

## Materials and Methods

### Study Population

**Figure 1:**
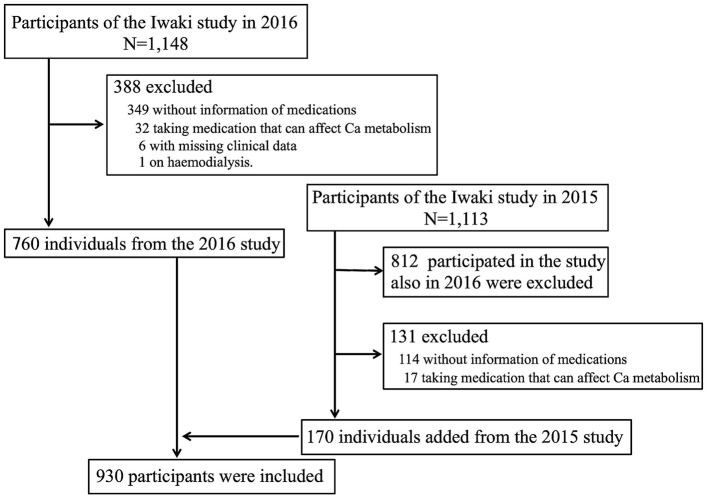
Subject selection flow diagram.

The design of the present study is cross-sectional, and participants were recruited from the Iwaki study, a health promotion study of Japanese people of over 20 years of age that aims to prevent lifestyle-related diseases and prolong lifespan. The Iwaki study is conducted annually in late May ~ early June in the Iwaki area of the city of Hirosaki in Aomori Prefecture in Japan (16, 17). Of the 1,148 individuals who participated in the Iwaki study in 2016, the following were excluded from the present study: 349 with missing information regarding use of medications, 32 who were taking medication that can affect Ca metabolism (Ca products, vitamin D, or a bisphosphonate), six with missing clinical data, and one who was undergoing haemodialysis. To increase the number of participants, 170 other individuals from the 2015 study cohort were added. Thus, a total of 930 participants (326 men and 604 women) aged 55.4 ± 15.9 years were included in the study (Figure 1).

This study was approved by the Ethics Committee of the Hirosaki University School of Medicine [No. 2014-377 (approved at April 22, 2014) and 2016-028 (approved at may 27, 2016)], and was conducted in accordance with the principles of the Declaration of Helsinki. Written informed consent was obtained from all the participants.

### Clinical and Laboratory Parameters

A fasting blood sample was collected in the morning from a peripheral vein of each participant while in a supine position for 5 min, after a 10 min rest in a sitting position. The following clinical parameters were measured: height, body mass, body mass index, percentage body fat, fasting blood glucose (FBG), glycated hemoglobin (HbA1c), systolic and diastolic blood pressure, serum concentrations of total cholesterol, triglyceride, high-density lipoprotein-cholesterol, uric acid, urea nitrogen, creatinine (Cr), Potassium (K), chloride (Cl), Ca, InP, intact PTH, and 25-dihydroxyvitamin D3 (25(OH)VitD3), and urinary albumin excretion (UAE). Percentage body fat was measured by bioimpedance using a Tanita MC-190 body composition analyser (Tanita Corp., Tokyo, Japan). HbA1c (%) is expressed as National Glycohemoglobin Standardization Program values. All laboratory tests were performed in a commercial laboratory (LSI Medience Co., Tokyo, Japan), in accordance with the manufacturers' instructions. Serum Ca concentration was corrected to serum albumin concentration, as previously reported (18). Estimated glomerular filtration rate (eGFR) was calculated using the following equations published by the Japanese Society of Nephrology: eGFR = 194 × Cr^−1.094^ × Age^−0.287^ for men and 194 × Cr^−1.094^ × Age^−0.287^ × 0.739 for women (19).

### Definitions

Vitamin D deficiency was defined by a serum 25(OH)VitD3 concentration <15 ng/ml. Diabetes was defined according to the 2010 Japan Diabetes Society criteria: FBG concentration ≥126 mg/dl (20), or for participants whose FBG was not measured, diabetes was defined by an HbA1c of ≥6.5%. None of the participants in the study was known to have type 1 diabetes. Hypertension was defined by a blood pressure ≥140/90 mmHg or current treatment for hypertension. Hyperlipidaemia was defined by a total cholesterol ≥220 mg/dl, TG ≥150 mg/dl, or current treatment for hyperlipidaemia.

### Statistical Analysis

Clinical parameters are given as mean ± standard deviations (SD) or, when the data were not normally distributed, as median with interquartile range (IQR). Relationships between serum intact PTH concentration and clinical parameters were assessed using linear regression analyses. Multiple linear regression analyses were used to evaluate the relationship between eGFR and serum intact PTH concentration, independent of factors shown to be associated with serum intact PTH concentration using univariable linear regression analysis (i.e., gender, age, height, percentage fat, FPG, total and HDL-cholesterol, Cl, Ca, InP, urea nitrogen, eGFR, UAE, Albumin, 25(OH)VitD3, and diastolic blood pressure). Furthermore, because the relationship between eGFR and serum intact PTH concentrations did not appear to be linear, smoothed spline curve fittings ωdgstλ of 100,000 were also applied for the analyses, and then the relationships were also evaluated after stratification according to eGFR (<60 *vs*. >60 ml/min/1.73 m^2^). Moreover, because there is a well-known association between vitamin D deficiency and a high serum concentration of intact PTH, we next evaluated the relationship between eGFR and serum intact PTH concentration after stratification according to serum 25(OH)VitD3 concentration (vitamin D deficient *vs*. non-vitamin D deficient). Serum Cr concentration was not included as a covariable for analyses using eGFR as a dependent variable, because it is used for the calculation of eGFR. *P* < 0.05 was regarded as indicating statistical significance. All analyses were performed using SPSS version 23.0 (IBM Japan, Tokyo, Japan) or JMP pro version 14.0 (SAS Institute Japan Ltd., Tokyo, Japan).

## Results

### Clinical Characteristics of the Study Subjects

The clinical characteristics of the participants are shown in Table 1. Their mean age was 55.4 years. Although most clinical characteristics appeared to be within the normal ranges (eGFR and the serum concentrations of Ca, InP, and intact PTH were 79.20 ± 15.76 ml/min/1.73m^2^, 9.50 ± 0.32 mg/dL, 3.51 ± 0.44 mg/dL, and 52.0 ± 20.2 pg/mL, respectively), the serum 25(OH)VitD3 concentration were slightly low [19.7 ± 7.7 ng/mL; individuals with 25(OH)VitD3 <15 ng/mL are considered to be vitamin D deficient (21)].

**Table 1:** Clinical characteristics of the participants.

**Characteristics**	
Gender (female)	326 (64.9)
Age (yr)	55.4 (15.9)
Height (cm)	159.7 (8.9)
Body weight (kg)	58.8 (11.1)
Fat (%)	27.1 (8.1)
Fasting plasma glucose (mg/dL)	91.1 (19.7)
HbA1c (%)	5.85 (0.58)
Total cholesterol (mg/dL)	204.9 (35.4)
Triglyceride (mg/dL)	92.9 (58.4)
HDL Cholesterol (mg/dL)	65.6 (17.0)
Potassium (mmol/L)	3.96 (0.31)
Chloride (mmol/L)	104.2 (2.0)
Calcium (mg/dL)	9.50 (0.32)
Inorganic phosphorus (mg/dL)	3.51 (0.44)
Urea nitrogen (mg/dL)	14.61 (4.49)
eGFR (ml/min/1.73 m^2^)	79.20 (15.76)
UAE (median (IQR)) (mg/gCre)	7.4 (4.5-15.0)
Uric Acid (mg/dL)	4.97 (1.34)
Albumin (g/dL)	4.49 (0.29)
Intact PTH (pg/mL)	52.0 (20.2)
25-dihydroxyvitamin D3 (ng/mL)	19.7 (7.7)
Systolic blood pressure (mmHg)	124.7 (18.3)
Diastolic blood pressure (mmHg)	74.9 (11.8)
Hypertension: *n* (%)	405 (43.5)
Hyperlipidemia: *n* (%)	429 (46.1)
Diabetes: *n* (%)	103 (11.1)

*Data are mean (SD) or number of subjects (%). eGFR, estimated glomerular filtration rate; UAE, urinary albumin excretion; PTH, parathyroid hormone; IQR, interquartile range*.

### Relationship Between eGFR and Serum Intact PTH Concentration

The relationships between clinical parameters and serum intact PTH concentration are shown in Table 2. Univariable analyses revealed correlations between serum intact PTH concentration and numerous clinical parameters (gender, age, height, percentage fat, FPG, total and HDL-cholesterol, Cl, Ca, InP, urea nitrogen, eGFR, UAE, Albumin, 25(OH)VitD3, and diastolic blood pressure). Multivariable correlation analysis with adjustment for these factors showed that the association between eGFR and serum intact PTH concentrations remained significant (β = −0.122, *p* < 0.001) (Table 2).

**Table 2:** Correlations between serum intact PTH concentration and other clinical parameters.

	**Univariable**		**Multivariable**	
**Characteristics**	**β**	***p***	**β**	***p***
Gender (female)	0.104	0.002**	0.051	0.314
Age (yr)	0.117	<0.001**	−0.034	0.436
Height (cm)	−0.094	0.004**	0.025	0.588
Body weight (kg)	−0.034	0.295	-	-
Fat (%)	0.109	<0.001**	0.039	0.301
Fasting plasma glucose (mg/dL)	0.261	<0.001**	0.049	0.163
HbA1c (%)	0.126	<0.001**	-	-
Total cholesterol (mg/dL)	0.182	<0.001**	0.040	0.224
Triglyceride (mg/dL)	0.093	0.005**	-	-
HDL Cholesterol (mg/dL)	0.119	<0.001**	0.139	<0.001**
Potassium (mmol/L)	−0.001	0.976	-	-
Chloride (mmol/L)	0.087	0.008**	0.083	0.008**
Calcium (mg/dL)	−0.136	<0.001**	−0.089	0.009**
Inorganic phosphorus (mg/dL)	−0.100	0.002**	−0.161	<0.001**
Urea nitrogen (mg/dL)	0.242	<0.001**	0.151	<0.001**
eGFR (ml/min/1.73 m^2^)	−0.211	<0.001**	−0.122	<0.001**
UAE (mg/gCre)	0.417	0.841	0.316	<0.001**
Uric Acid (mg/dL)	0.006	0.841	-	-
Albumin (g/dL)	−0.204	<0.001**	–0.042	0.218
25-dihydroxyvitamin D3 (ng/mL)	−0.189	<0.001**	−0.236	<0.001**
Systolic blood pressure (mmHg)	0.102	0.002**	-	-
Diastolic blood pressure (mmHg)	0.137	<0.001**	0.137	<0.001**
Hypertension: *n* (%)	0.102	0.002**	-	-
Hyperlipidemia: *n* (%)	0.053	0.105	-	-
Diabetes: *n* (%)	0.004	0.915	-	-

P < 0.05 and <0.01 are indicated by * and **, respectively. Data are mean ± SD or number of subjects (%). eGFR, estimated glomerular filtration rate; UAE, urinary albumin excretion; InP, inorganic phosphorus; PTH, parathyroid hormone.

### Serum 25(OH)VitD3 Concentration-Dependent Association Between eGFR and Serum Intact PTH Concentration

**Figure 2:**
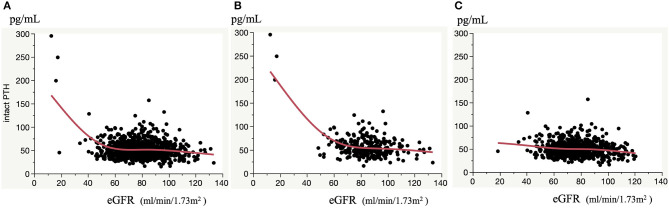
Correlation between eGFR and serum intact PTH concentrations. Correlation in all participants **(A)**, and participants with vitamin D deficiency **(B)** and non-vitamin D deficiency **(C)**. Smoothed spline curves are shown in each panel.

As shown in Table 2, the association between eGFR and serum intact PTH concentration was weaker than the association between UAE or serum 25(OH)VitD3 concentration and serum intact PTH concentration (β = 0.316, *p* < 0.001 and β = −0.236, *p* < 0.001, respectively). However, the association between eGFR and serum intact PTH concentration became much stronger when the participants were stratified according to eGFR or serum 25(OH)VitD3 concentration. Namely, the relationship between eGFR and serum intact PTH concentration did not appear to be linear, and therefore a smoothed spline curve was applied, which demonstrated a biphasic correlation with a division at an eGFR of ~60 mL/min/1.73 m^2^, below which the correlation coefficient was higher (β = −0.405, *p* < 0.001) (Figure 1 and Table 3). Next, because there is a well-known association between vitamin D deficiency and a high serum intact PTH concentration (13–15), we evaluated the relationship between eGFR and serum intact PTH concentration after stratification according to serum 25(OH)VitD3 concentration, which was used to assess the vitamin D status of each individual. Serum 25(OH)VitD3 concentrations have been shown to be low in East Asian populations (22), and, indeed a large percentage of the participants in the present study were classified as vitamin D deficient (29.4%). A correlation between eGFR and serum intact PTH concentration was identified only in participants with vitamin D deficiency according to this definition [(β = −0.154, *p* = 0.012] (Table 3). Furthermore, a regression analysis with smoothed spline curve fitting showed a biphasic correlation between eGFR and serum intact PTH concentration only in those participants with vitamin D deficiency (Figure 2).

**Table 3:** Correlations between eGFR and intact PTH in participants stratified according to eGFR and serum vitamin D concentration.

	**eGFR**				**Vitamin D**			
	**<60**		**>****60**		**<15**		**>****15**	
	**β**	***p***	**β**	***p***	**β**	***p***	**β**	***p***
Univariate	−0.665	<0.001**	−0.081	0.020*	−0.419	<0.001**	−0.126	0.001**
Adjusted #	−0.405	<0.001**	−0.038	0.311	−0.154	0.012*	−0.080	0.075

P < 0.05 and <0.01 are indicated by * and **, respectively. The data were adjusted for gender, age, height, percentage fat, FPG, total and HDL-cholesterol, Cl, Ca, InP, urea nitrogen, eGFR, UAE, Albumin, 25(OH)VitD3, and diastolic blood pressure. 25(OH)VitD3: 25-dihydroxyvitamin D3; UAE, urinary albumin excretion; InP, inorganic phosphorus; eGFR, estimated glomerular filtration rate.

Taken together, these results indicate that a reduction in eGFR represents a significant risk for an increase in serum PTH concentration only when eGFR is <60 and vitamin D deficiency is present in a general Japanese population.

## Discussion

In this cross-sectional study of a general Japanese population, we found that a reduction in eGFR is a significant risk factor for an increase in serum intact PTH concentration, but only when vitamin D is deficient. Furthermore, the relationship appears to be biphasic, with a division at an eGFR of ~60 mL/min/1.73 m^2^, and below this it is much stronger (β = −0.405, *p* < 0.001), with a correlation coefficient higher than those describing the relationships between UAE or serum 25(OH)VitD3 concentration and serum intact PTH concentration (β = 0.181, *p* = 0.123 and β = −0.118, *p* = 0.132, respectively), which were the strongest across the entire sample. Thus, it seems that when eGFR is <60 mL/min/1.73 m^2^, a reduction in eGFR substantially increases serum intact PTH concentration only in the presence of vitamin D deficiency in the general population. The fact indicates that individuals with an eGFR <60 mL/min/1.73 m^2^ are at risk for hyperparathyroidism secondary to a reduction in eGFR only in the presence of vitamin D deficiency.

Vitamin D supplementation can be recommended to anyone who is vitamin D deficient, because a number of observational studies have shown that it is associated with higher mortality from non-skeletal chronic diseases, such as cancer and cardiovascular disease (23–26). In addition to its effects on these diseases, it is also likely to have a beneficial effect in CKD-MBD, because the active form of vitamin D, 1,25(OH)2VitD3, suppresses serum PTH concentration both directly and indirectly, through the vitamin D receptor in cells of the parathyroid gland and an increase in serum Ca, respectively (14). Furthermore, vitamin D concentrations have been shown to be relatively low in East Asian populations, and patients with CKD-BMD are likely to be vitamin D-deficient (27). Therefore, the evaluation of vitamin D status is important in patients with CKD, especially if they are East Asian, in order to identify individuals that might benefit from vitamin D supplementation. However, the present findings suggest that such an evaluation may be less effective for patients in a standard clinical setting. Instead, we recommend measuring serum 25(OH)VitD3 concentration when the eGFR is <60 mL/min/1.73 m^2^, and if the patient is deficient, supplementation of vitamin D should be considered to mitigate secondary hyperparathyroidism leading to CKD-MBD.

The vitamin D status-dependent association between eGFR and serum intact PTH concentration identified here is novel and worthy of attention. Although the relationship between eGFR and serum intact PTH concentrations has been evaluated previously, none of the previous studies did so with reference to vitamin D status, and therefore the difference in methodology may lead to findings different from the previous ones. Interestingly, a previous study of a general population showed that intact PTH concentration began to increase at an eGFR of ~45 (28), which is consistent with our result obtained using the entire sample (Figure 1). However, the present study has added to this by stratifying the participants on the basis of their vitamin D status, finding that intact PTH begins to increase at a higher eGFR of ~60 mL/min/1.73 m^2^ in the presence of vitamin D deficiency, but the increases in PTH were not substantial at any eGFR if vitamin D was not deficient. Thus, our results indicate that associations between eGFR and serum intact PTH concentration that do not consider vitamin D status should be interpreted with caution. Because intact PTH concentration may begin to increase at an earlier stage of CKD in the presence of vitamin D deficiency, vitamin D status should be evaluated, even in the early stages of CKD, to mitigate hyperparathyroidism developing secondary to CKD or CKD-MBD.

Serum Ca and phosphate concentrations are tightly regulated. PTH, vitamin D, and serum ionized Ca itself co-ordinately regulate Ca transport in the gut, kidney, and bone (27); therefore, defects in any of these mechanisms might affect the serum concentration of Ca or phosphate. Thus, when associations of any of these parameters with any of the factors described above are evaluated, those factors should also be included together as possible confounders. In the present study, we evaluated the relationship between eGFR and serum intact PTH concentration, with adjustment for the serum concentrations of InP, Ca, and 25(OH)VitD3, and therefore the association reported here does not merely reflect an abnormality in mineral metabolism overall. Further, adjustment with vitamin D seems to be also important, because vitamin D has been shown to prevent kidney damage and a reduction in GFR in experimental models through a number of mechanisms, including activation of the renin-angiotensin-aldosterone system, reduction in the production of inflammatory mediators, promotion of podocyte survival, and reduction of albuminuria and glomerulosclerosis (28–34). Moreover, lower serum Ca concentration has been reported to be a significant risk factor for a rapid decrease in eGFR, independent of the previously reported metabolic risk factors for kidney dysfunction, such as blood pressure, dyslipidaemia, and diabetes, even when they are within their normal physiological ranges (35). Therefore, the association between eGFR and serum intact PTH concentration observed independent of any of the expected confounding factors seems to be biologically plausible.

We here found vitamin D status-dependent association between eGFR and serum intact PTH concentration, which indicates that measurements of vitamin D or intact PTH concentrations alone may have limited impact on evaluating individuals at risk for CKD-MBD. Further, as shown, correlation between vitamin D and serum PTH concentrations is not substantial, and therefore correction of low vitamin D concentrations frequently does not correct PTH concentrations and then active vitamin D supplementation was required to correct PTH concentrations (36, 37). Moreover, the clinical significance of correcting serum PTH concentrations does not seem to be clearly shown, and therefore measurements of serum intact PTH concentrations in general population need to be evaluated further in the future. Fibroblast growth factor-23 (FGF23), which is produced predominantly by osteoblasts and osteocytes, regulates renal tubular phosphate reabsorption, acts as a counter-regulatory hormone of active vitamin D, increases as a consequence of a decreased GFR, and thus plays a pivotal role in CKD-MBD (37, 38). Since serum FGF23 concentrations can be a better indicator representing pathological background of CKD-MBD than serum PTH concentrations, using serum FGF23 concentration instead of serum PTH concentrations as the dependent variable may provide more definitive results. However, we did not measure serum FGF23 concentrations, and therefore the issue remains to be evaluated.

The threshold to define vitamin D deficiency has no consensus world wide, as it can be defined variously according to population-based reference limits for serum 25(OH)VitD3 concentrations or biological indices, such as hypocalcemia, and elevated intact PTH concentrations (37). Further, extent of sunshine exposure and amount of Ca consumed surely affect serum 25(OH)VitD3 concentrations (14). In Japan, a serum 25(OH)VitD3 concentration <20 ng/ml was set as the threshold by major academic societies such as the Japan Endocrine Society (JES) (39). However, the JES also set a serum 25(OH)VitD3 concentration <15 ng/ml to be suggestive of the diagnosis of hypocalcemia due to vitamin D deficiency (38). Further, the Pediatric Endocrine Society recommends a serum 25(OH)VitD3 concentrations of <20 ng/ml and <15 ng/ml for the thresholds to define vitamin D insufficiency and deficiency, respectively (40). Moreover, as serum 25(OH)VitD3 concentrations have been shown to be low in East Asian populations (22), more than half, 54.6%, of the participants in the present study can be classified as vitamin D deficient if vitamin D deficiency was defined using a threshold of serum 25(OH)VitD3 concentration <20 ng/mL. Thus, far, for this study, we defined vitamin D deficiency by a serum 25(OH)VitD3 concentration <15 ng/ml. Accordingly, the findings observed here may be specific to the Japanese population, and therefore further studies should be conducted also in other ethnicities.

The present study had several strengths and limitations. The strengths included the fact that statistical adjustments were made for multiple factors that could have confounded the results and that a general population-based sample was studied. Furthermore, patients taking medication that affects Ca metabolism were excluded, because these drugs affect the serum concentrations of Ca, 25(OH)VitD3, and intact PTH. The limitations included the fact that the participants were selected from a health promotion study and not from a population undergoing health checks, which implies that the participants may have been more invested in their health than the general population. Furthermore, because the participants were recruited at population-based health care examinations, few of them had CKD stages 3 or 4 or eGFR <60 (*n* = 89), and therefore the statistical power of the analyses may not have been sufficient, especially when the participants were stratified on the basis of their eGFR. However, given that significant results were obtained, this limitation does not appear to be substantial.

In conclusion, individuals with an eGFR of <60 mL/min/1.73 m^2^ seem to be at risk of hyperparathyroidism secondary to a reduction in eGFR only if they are vitamin D deficient at least in a Japanese population. These results imply that serum 25(OH)VitD3 concentration should be measured when eGFR is <60 mL/min/1.73 m^2^, to identify individuals who might benefit from PTH suppression therapy for secondary hyperparathyroidism and CKD-MBD as well as vitamin D supplementation, although further large-scale studies, in particular of individuals with CKD stages 3 or 4, are warranted.

## Data Availability Statement

All datasets generated for this study are included in the article/supplementary material.

## Ethics Statement

The studies involving human participants were reviewed and approved by The Ethics Committee of the Hirosaki University School of Medicine. The patients/participants provided their written informed consent to participate in this study.

## Author Contributions

MD, HM, IT, KS, and KI designed the study. MD analyzed, interpreted the data and wrote the manuscript. TF, MM, SM, YN, JT, YM, MY, IT, KS, and KI contributed to data acquisition. SM, JT, and HM contributed to data interpretation. IT, KS, and KI take responsibility for the integrity and the accuracy of the data. All authors reviewed and edited manuscript, and have approved the final version of the manuscript.

## Conflict of Interest

The authors declare that the research was conducted in the absence of any commercial or financial relationships that could be construed as a potential conflict of interest.
